# Nanostructural control of methane release in kerogen and its implications to wellbore production decline

**DOI:** 10.1038/srep28053

**Published:** 2016-06-16

**Authors:** Tuan Anh Ho, Louise J. Criscenti, Yifeng Wang

**Affiliations:** 1 Geochemistry Department, Sandia National Laboratories, Albuquerque, New Mexico 87185, USA; 2 Nuclear Waste Disposal Research and Analysis Department, Sandia National Laboratories, Albuquerque, New Mexico 87185, USA

## Abstract

Despite massive success of shale gas production in the US in the last few decades there are still major concerns with the steep decline in wellbore production and the large uncertainty in a long-term projection of decline curves. A reliable projection must rely on a mechanistic understanding of methane release in shale matrix–a limiting step in shale gas extraction. Using molecular simulations, we here show that methane release in nanoporous kerogen matrix is characterized by fast release of pressurized free gas (accounting for ~30–47% recovery) followed by slow release of adsorbed gas as the gas pressure decreases. The first stage is driven by the gas pressure gradient while the second stage is controlled by gas desorption and diffusion. We further show that diffusion of all methane in nanoporous kerogen behaves differently from the bulk phase, with much smaller diffusion coefficients. The MD simulations also indicate that a significant fraction (3–35%) of methane deposited in kerogen can potentially become trapped in isolated nanopores and thus not recoverable. Our results shed a new light on mechanistic understanding gas release and production decline in unconventional reservoirs. The long-term production decline appears controlled by the second stage of gas release.

Sucessful gas production from shales in the United States in the last decade has initiated interest around the world^1^. Europe, Australia, and Africa are now starting to evaluate and explore their unconventional reservoirs. The reserves and economic feasibility of shale gas as an economic energy source depends on Estimated Ultimate Recoveries (EUR) based on the production history of current plays^2^. The current production data generally indicate a steep decline in productivity over the first 3 years (i.e., the output of a typical well drops 80–90%)^3^,^4^,^5^. This raises serious concerns about the long-term sustainability of shale gas production^5^. Maximizing the production rate and extending the wellbore life-time are important to the shale gas industry.

Another major concern of the shale gas revolution is that the use of a few years of production history to predict decades of commercial production may have overestimated productivity^2^,^5^,^6^. Among many other methods, a decline curve analysis is the most frequently used method for EUR estimation^4^,^7^. Decline curve analysis is performed using the empirical Arps’ equation^8^


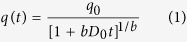


where *q(t)* and *q*_*0*_ are the gas production rates at time *t* and *t* = 0, respectively. *D*_*0*_ is the decline rate at *t* = *0* and *b* is the constant that controls the curvature of the decline trend. The selection of a wrong *b* value would have a tremendous impact on reserve estimation, particularly when *b* is too high^9^. In equation [1], if 0 < *b* < 1 the cumulative production is finite and if b ≥ 1 cumulative production is infinite, which is unreasonable^7^. However, many have reported that *b* > 1 yields the best fit to US shale production data^4^,^7^,^10^. For example, Baihly *et al*.^4^ reported *b* ranging from 0.637 to 1.694 for multiple shales. Because using *b* > 1 will tremendously increase EUR, many have warned that the use of a few years of historical production data to forecast the future of shale might be incorrect^2^,^5^,^11^. Indeed, many found that, as more production data become available *b* tends to decrease^4^,^7^,^10^. Predicting and using the right *b* constant is therefore important in analyzing the future of shale production.

Shales are characterized with extremely low permeabilities (1–100 nanodarcy) and with the predominant presence of nanometer-sized pores (1–200 nm)^12^. Whether a shale formation can be targeted for oil and gas exploration depends largely on the amount and type of organic content^3^,^13^,^14^. Usually, the higher the concentration of organic matter in rock, the better its source potential^2^. Organic matter can adsorb gas and store significant amounts of free gas in its pores (e.g., Barnett Shale)^15^. Data in the literature also suggests that clay minerals do not significantly contribute to methane sorption in organic-rich shale (e.g., Barnett, Haynesville)^16^,^17^. Under high pressure and temperature, organic matter is usually transformed into kerogen during sediment diagenesis^13^,^18^. Based on the atomic H/C and O/C ratios (i.e., van Krevelen diagram), kerogen is usually classified into three types^18^. Type I (e.g., Green River kerogen) is primarily formed in lacustrine and sometimes marine environments. It is highly aliphatic with H/C >1.5 and O/C from 0.03 to 0.1. Type II (e.g., Barnett kerogen) is typically formed in deep marine environments. It is rich in hydrogen and low in carbon (H/C ~ 1.3, O/C ~ 0.15). Sulfur is also associated with this type of kerogen. Type III is derived from higher plant debris (e.g., coal). It has H/C <0.8 and O/C from 0.03 to 0.3.

Horizontal drilling and hydraulic fracturing are two innovative techniques that enable commercial exploration of natural gas from impermeable shale formations^19^. The former enhances the extraction volume. The latter enhances shale permeability by creating a fracture network through an injection of a pressurized fluid into a wellbore. After a stimulation, gas is released from the shale matrix and migrates to the created fractures and then to a production wellbore. Because flow rates are usually high in the induced fractures, gas migration from the low permeability shale matrix into the fractures is the time-limiting step that controls production rate^4^. Thus, understanding the gas extraction process from the nanopores is crucial to explaining the field-observed production decline.

In this work we performed molecular dynamics (MD) and Monte Carlo (MC) simulations using LAMMPS^20^ to investigate methane adsorption to type II post-mature kerogen, methane extraction from nanopores, and the properties of methane in nanoporous kerogen. There are several advantages of our simulations compared to others. First, we used realistic kerogen models. Many used simple carbon porous material as a model for kerogen^21^,^22^,^23^,^24^. These simple models ignore chemical details, and, in some cases, the complexity and heterogeneity of the kerogen (e.g. by using a slit-shape pore). Thus, they cannot accurately capture the physical and chemical properties of complex kerogen^25^,^26^. Second, we conducted extraction simulations under reservoir-relevant conditions. In our simulations, methane was withdrawn from the porous structure by defining an extracting region. When a molecule of gas flows into that region, it is withdrawn from the system by deletion. This allows us to study the flow of methane in porous materials as the gas pressure gradually decreases over time because of extraction. This also eliminates a serious problem encountered in other non-equilibrium MD simulations in which large, unrealistic forces are usually used to create flow^27^,^28^,^29^. Our results indicate that the extraction rate decreases rapidly in an early stage of extraction, in some sense, resembling field observations. Our work provides a microscopic view of methane extraction from a heterogeneous nanoporous kerogen matrix and may shed a light on our mechanistic understanding of the overall gas extraction process in the field.

## Results

### Model construction of kerogen

**Figure 1:**
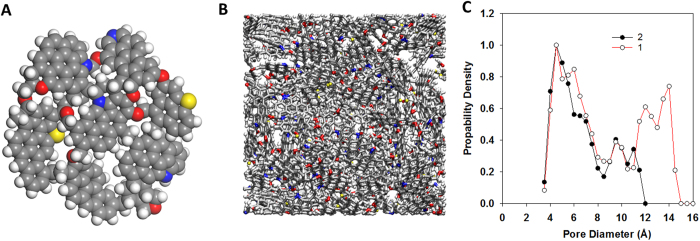
Post-mature kerogen molecule used in our simulations (**A**). Red, blue, yellow, grey, and white spheres represent oxygen, nitrogen, sulfur, carbon, and hydrogen atoms, respectively. A representative condensed kerogen sample at ambient conditions was obtained by conducting a series of MD simulations as described in the Methods section (**B**). Pore size distributions of two extreme kerogen samples collected in MD simulations: 1 (red) and 2 (black) (**C**).

The kerogen model (Fig. 1A) used in our work was developed by Ungerer *et al*.^30^ to reproduce the elemental and functional analysis data of kerogen by Kelemen *et al*.^26^. It is representative of over-mature kerogen (Type II-D) found in the Duvernay organic-rich marine shale formation and similar to that found in Barnett shale. In the work of Ungerer *et al*.^29^,^30^,^31^, the kerogen molecule was modeled by implementing the PCFF+ force field^32^, which describes atomic dispersion-repulsion interactions using the Lennard Jones 6–9 potential. This makes it difficult to simulate kerogen with other constituents (e.g., water, clays, CO_2_, hydrocarbon) which are often simulated using force fields that implement the Lennard Jones 6–12 potential to describe atomic dispersion and repulsion. In our simulations, kerogen was simulated using the CVFF force field^33^. As we show below the condensed kerogen density, pore size distribution, and methane adsorption isotherm are not only comparable with those obtained for kerogen modeled by using the PCFF+ force field but also with experimental results.

Condensed kerogen (Fig. 1B) was created by conducting a series of MD simulations as described in the Methods section. At ambient conditions, the density of our kerogen model ranges from 1.172 g/cm^3^ (Sample 1) to 1.287 g/cm^3^ (Sample 2). These two samples serve as two bounding cases that we will use to report results in this paper. The other samples we studied fall between these two in terms of density, pore-size distributions, and gas adsorption. The average density of kerogen calculated from the 9 collected samples is 1.22 ± 0.04 g/cm^3^. This density is consistent with that determined using the PCFF+ force field^30^. When compared with experimental data, our calculated density is in good agreement with that reported by Stankiewicz *et al*.^34^ for the kerogen in the Duvernay shale (1.28 ± 0.3 g/cm^3^).

In Fig. 1C, we report the pore size distributions (PSD) of the two kerogen samples. The pore size distribution was calculated by applying the method proposed by Gubbin *et al*.^35^ using an argon probe (PSD calculated using He probe is insignificantly different from PSD calculated using argon probe, data not shown for brevity). The pore size varies from 4 to 15 Å for Sample 1 and from 4 to 12 Å for Sample 2. The differences observed in the density and the PSD of the different kerogen samples represent the heterogeneity of shale formations^16^. The PSD indicates that only micropores (<2 nm^36^) are observed in our models. Mesopores (2–50 nm) and macropores (>50 nm)^36^ are not present in our simulations due to limitations in our system size. Experimentally, PSD is studied using mercury porosimetry and low-pressure gas adsorption analyses. Because mercury porosimetry cannot detect micropores, our results need to be compared with those measured using gas adsorption techniques. Direct comparison between our calculated PSD and experimental data for isolated kerogen cannot be made because, to our knowledge, this data is not available. However, comparison with experimental data for shale (i.e., including organic and inorganic matter) indicate that, except for the absence of mesopores and macropores, the PSD for the micropore size fraction is in good agreement with those found for the Barnett^37^, Alum-Denmark^38^, and numerous other shales in the United States such as the Haynesville, Marcellus, and Woodford^39^.

### Adsorption isotherm of methane on kerogen

**Figure 2:**
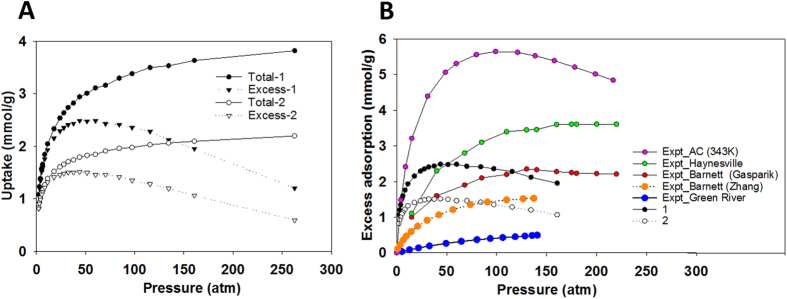
Total uptake (circles) and excess adsorption (triangles) of methane on kerogen samples 1 (black) and 2 (white) at 338 K as a function of pressure (**A**). Comparison of the excess adsorption for sample 1 (black circles) and 2 (white circles) with experimental results for activated carbon (purple circles), Haynesville shale (green circles), Barnett shale (red circles by Gasparik *et al*.^16^, and orange circles by Zhang *et al*.^40^), and Green River kerogen (blue circles) (**B**). Green River kerogen is type I low thermal maturity kerogen. The excess adsorption data for the Barnett and Haynesville shales are measured for the whole shale (i.e., including organic and inorganic matter) and normalized by the total organic carbon. The comparison suggests that excess adsorption calculated for isolated kerogen can provide approximate values for methane adsorption in organic-rich shales.

In Fig. 2A we present the total uptake and excess adsorption of methane obtained for the two bounding kerogen samples. To calculate total uptake (i.e., total amount of methane in simulation box) we conducted grand-canonical Monte Carlo (GCMC) simulations as described in the Methods. Excess adsorption is the difference between the total uptake and the amount of gas in the free volume in kerogen pores (see Methods). The results indicate that when pressure increases the total uptake increases rapidly at low pressure and slowly at high pressure. Comparison between total uptake and excess adsorption suggests that gas present in kerogen at low pressure is mainly adsorbed gas (i.e., the total uptake and the excess adsorption are the same). At high pressure, more gas fills into the free volume in the center of kerogen nanopores, resulting in a large difference between the total uptake and the excess adsorption. This observation is in good agreement with numerous simulations and experimental results for gas adsorption on metal organic frameworks^41^, carbon materials^24^, shale and coal^42^,^43^.

In Fig. 2B we compare our excess adsorption with experimental results from the literature such as data for Barnett (total organic carbon TOC = 3.5%, VR = 2.2%) and Haynesville (TOC = 3.3%, VR = 2.1%) shales by Gasparik *et al*.^16^, data for kerogen from Green River (VR = 0.56) and organic-rich Barnett shale (TOC = 6.6%, VR = 2.01) by Zhang *et al*.^40^ Barnett and Haynesville are the two organic-rich shales that have similar maturity to our kerogen model and are within the dry gas generation window^39^. These experimental data were measured for the whole shale (i.e., including organic and inorganic matter) and normalized by the TOC. Note that the experimental results for Barnett shales (red and orange circles) are from two groups (Gasparik *et al*. and Zhang *et al*.). The fact that two different samples from Barnett shales or two samples of similar TOC and VR (Barnett and Haynesville) exhibit different excess adsorption data demonstrates the diversity and heterogeneity of shales, consistent with our simulations (sample 1 and 2). Green River kerogen is type I kerogen with the vitrinite reflectance (VR) of 0.56% indicating low thermal maturity. The comparison indicates that the excess adsorption obtained for the simulated kerogen is higher than the measurement for Green River kerogen, probably due to the effect of thermal maturity (i.e., the Green River kerogen is less mature than the model kerogen). The comparisons also suggest that our excess adsorption data is of the same order of magnitude with actual measurements on Barnett and Haynesville shales. Thus, excess adsorption calculated for isolated kerogen can provide approximate values for methane excess adsorption in organic-rich shales. This is in a good agreement with the conclusion that organic matter plays a dominant role in gas adsorption in organic-rich shales. Other shale components such as clay minerals are reported to not contribute significantly to methane sorption in organic-rich shales^16^. Heller and Zoback^17^ investigated methane sorption on pure illite, kaolinite, and activated carbon (a proxy for kerogen) and concluded that the amount of gas adsorbed on carbon is three orders of magnitude higher than that on clay minerals.

However, the comparison also illustrates a difference between our results and those measured on Barnett and Haynesville shales. While our excess adsorption data show a clear maximum at low pressure (0–50 atm), experimental data for shales do not exhibit a clear maximum. This difference could be attributed to several factors. First, the experimental data were measured for the whole shale and normalized by TOC. Inorganic matter might affect the pore size, the pore connectivity, and the interaction of methane with pores. Second, in the GCMC technique used to calculate our adsorption data, methane molecules can be inserted into isolated pores while in experiment methane can access only the connected pores. We also compared our excess adsorption with experimental results for methane onto activated carbon^44^, which is a proxy for kerogen^17^. Both our simulated results for kerogen and the experimental data for activated carbon^44^ exhibit a distinct maximum in excess adsorption, but methane excess adsorption in activated carbon is much higher. This difference raises questions regarding the use of porous carbon materials as surrogates for kerogen.

### Extraction of methane from nanopores in kerogen

**Figure 3:**
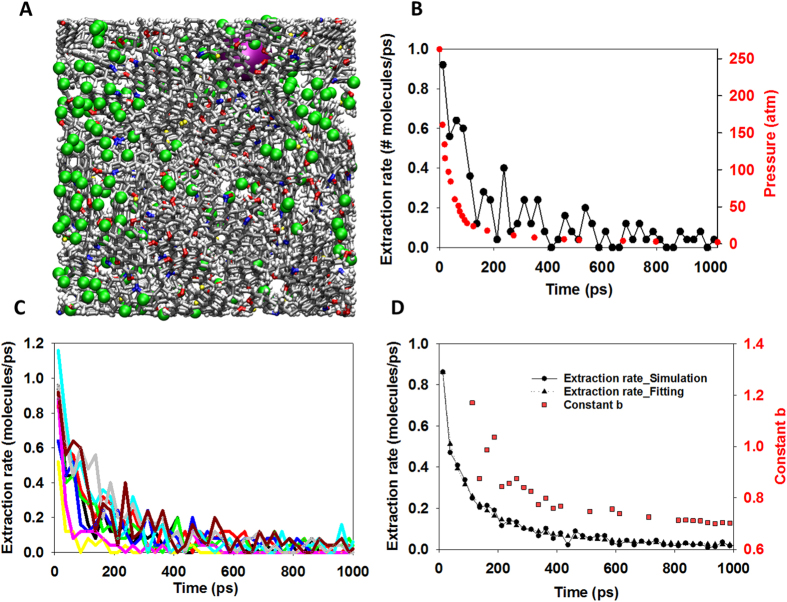
Snapshot demonstrates the method used to extract methane molecules (green spheres) from kerogen sample 1 (**A**). When a methane molecule moves into a defined region (purple sphere) we extract it from the system by deleting that molecule. Extraction rate (black circles-left y axis) and pressure (red circles-right y axis) are plotted as a function of time during the extraction for sample 1 (**B**). The extraction rate is calculated for 9 kerogen samples (**C**). The averaged extraction rate is calculated from those obtained for 9 samples (black circles-left y axis) and the fitted curve is calculated using decline curve analysis (black triangles-left y axis) with b = 0.7022 (**D**). Constant *b* is obtained when fitting equation [1] with simulation data at different times (red squares-right y axis) (**D**).

To study the flow of methane in complex kerogen structures we conducted an extraction simulation (Fig. 3A) mimicking the field production where natural gas is withdrawn from a reservoir. In our simulation, methane is withdrawn from the porous structure by deleting the methane molecules that move into a defined region (large purple sphere) (see Methods for more details). Because of the pressure gradient from the methane-filled kerogen to the extraction region, methane molecules in the kerogen pores diffuse into the extraction region if they are not trapped in isolated pores. This method allows us to study (i) the flow of methane in porous materials as the system pressure gradually decreases because of methane removal, (ii) the extraction rate as a function of time, (iii) the effect of adsorption, desorption, diffusion and nanoporous structure on the extraction rate. This method also eliminates one of the most serious troubles encountered in other non-equilibrium MD simulations in which large, unrealistic forces are usally used to induce flow^27^,^28^,^29^.

In Fig. 3C, we report the methane extraction rates for 9 kerogen samples as a function of time. The results indicate that the rate significantly decreases in a short period of time after initiating extraction. Figures 3B and S1 show that we found a good correlation between the extraction rate and pressure of the system. The pressure also steeply decreases after initiating extraction. Afterwards, the pressure decreases slowly as a function of time. At the turning point (i.e., pressure ~17 atm), where the pressure and extraction rate start to decrease more slowly, 47 and 30% of methane were recovered from kerogen samples 1 and 2, respectively. After this point, both pressure and extraction rate decrease gradually. More methane is extracted until after 16 ns, 50% of the methane is recovered for sample 1 and after 26 ns, 35% of the methane is recovered for sample 2. At these times, there is still 3 and 35% of methane remaining in the kerogen samples (1 and 2, respectively) that is trapped in isolated pores. These results indicate that the pores in sample 1 are better connected than those in sample 2 and that the ultimate recovery depends on network connectivity. If we exclude the methane in the isolated pores about 50% of the methane is recovered quickly and 50% is recovered slowly.

The reason for the rapid decrease in extraction rate is shown in Fig. 2A. As shown in the figure, the total uptake and excess adsorption curves (for both samples) start to separate from each other at the pressure of ~17atm, which is the turning point observed in the extraction rate and pressure curves in Fig. 3B, suggesting that, at the early stage of extraction (i.e., at high pressures), the methane molecules withdrawn from kerogen are mainly present as pressurized free gas in the center of the nanopores. At pressures above ~17atm, where there is a significant difference between total uptake and excess adsorption (Fig. 2A), the extraction rate and pressure drop significantly (Fig. 3B,C). The main driving force for methane flow into the extraction region is the pressure gradient created by methane removal. At pressures below ~17atm, when the total uptake and excess adsorption are the same, there is little methane in the free volume. Beyond this point, most of the methane molecules extracted are adsorbed. The pressure gradient is no longer the main factor controlling methane migration. The extraction rate is much slower as it is determined by the desorption and diffusion rates.

### Properties of methane confined in kerogen nanopores

**Figure 4:**
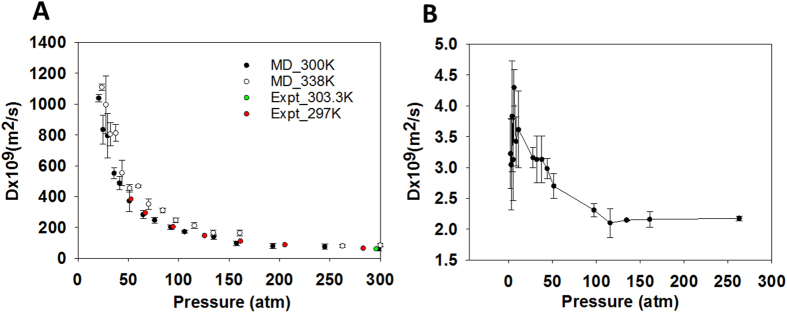
Self-diffusion coefficient D of bulk methane as a function of pressure (**A**). Self-diffusion coefficient of bulk methane was studied by conducting MD simulations for 300 methane molecules using NPT ensemble at the temperature of 300 K (black circles) and 338 K (white circles). The pressure ranges from 2 to 300 atm. The results at 300 K are compared with experimental data (red and green circles). Self-diffusion coefficient of all methane (i.e., including free gas and adsorbed gas) inside kerogen sample 1 as a function of pressure during the extraction (**B**). This result was obtained by running MD simulations using NVT ensemble (338 K) starting from the configurations at different pressures obtained during the extraction process as described in Fig. 3A. All simulations were conducted for 40 ns. The last 30 ns trajectory was divided into 3 blocks of 10 ns each to calculate diffusion coefficient and error. Compared with that of bulk methane, the diffusion coefficient of methane inside kerogen is much smaller (up to 3 orders of magnitude smaller), and furthermore it does not change significantly as a function of pressure.

It is known that chemical species confined in nanopores can behave significantly differently from that in a bulk system^12^. In Fig. 4 we compare the dynamic properties of bulk methane with methane confined in kerogen. In Fig. 4A we report the self-diffusion coefficient (D) of bulk methane as a function of pressure at 300 and 338 K. The results at 300 K are compared with experimental data around 300 K^45^,^46^. The comparison illustrates excellent agreement between computational and experimental results. Our results also indicate that the self-diffusion coefficient of bulk methane exponentially decreases up to two orders of magnitude when the pressure increases from 20 to 300 atm.

In Fig. 4B, we show the diffusion coefficient of all methane (i.e., including free gas and adsorbed gas) confined in kerogen sample 1 (the same result for sample 2 is reported in Figure S2 of the supporting information) at different pressures. We observed that the diffusion coefficient of methane in kerogen sample 1 is higher than that of methane in sample 2. This observation agrees with the previous conclusion that pores in sample 1 are better connected than those found in sample 2. Compared with bulk methane reported in Fig. 4A, the methane inside kerogen diffuses much more slowly (up to 3 and 5 orders of magnitude slower for sample 1 and 2, respectively). Our results also indicate that the diffusion coefficient of methane inside kerogen does not change significantly during extraction (i.e., as pressure decreases), in a striking contrast to bulk methane. This suggests that nano-confinement, adsorption, and pore connectivity play key roles in determining methane extraction rates. As the diffusion of methane in kerogen is always slow, the movement of methane into the extraction region at high pressure as discussed in Fig. 3 is governed by the pressure drop. The diffusion plays a small role at high pressures at which the number of methane molecules present as free gas is abundant. However, at low pressure there is little free gas, the governing factor is either diffusion or desorption. After the turning point shown in Fig. 3B, there is still plenty of methane remaining in the connected pores in the kerogen samples, but the extraction rate is very slow.

**Figure 5:**
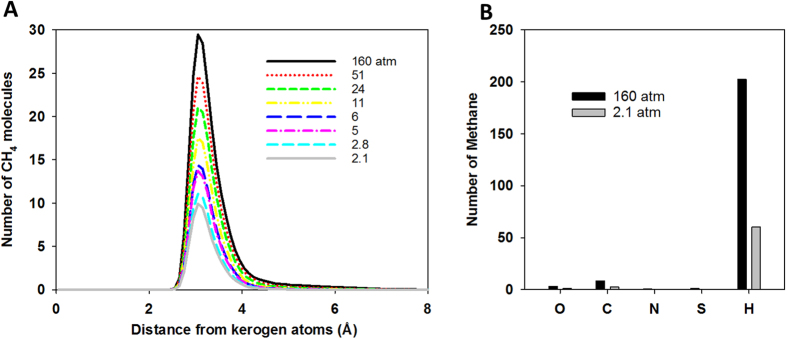
Profile of number of methane molecules as a function of distance from kerogen atoms at different pressure (**A**). Number of methane molecules associated with specific kerogen atom types at 160 atm (black) and 2.1 atm (grey) (**B**). These data were calculated for sample 1.

In Fig. 5A we report the profile of the number of methane molecules for sample 1 as a function of distance from kerogen atoms for a range of pressures. At high pressures, the peak of the profile is high and broad. At low pressures, the peak is low and narrow. The broad peak indicates that methane molecules are located both close to and far from the kerogen atoms, suggesting the existence of both adsorbed and free gas. As more gas is extracted, the number of free methane molecules decreases, and methane is mainly located near the kerogen atoms. At 2.1 atm, methane molecules concentrate only 2.5 to 4 Å away from the kerogen atoms. The number of methane molecules associated with specific atom types in kerogen is shown in Fig. 5B. The majority of methane molecules are located near the hydrogen atoms of kerogen.

## Discussion

In summary, using molecular simulations, we studied methane disposition and release in the nanoporous kerogen matrix. The simulations reveal two stages of methane release from kerogen nanopores, each with a distinct release mechanism. At the early stage of gas extraction, when the gas pressure is high, methane molecules withdrawn from the system are mainly pressurized free gas, and the migration of methane is driven by the gas pressure gradient. At the late stage, when the gas pressure is low, gas molecules extracted from the system are adsorbed gas. Methane desorption coupled with diffusion becomes the time-limiting step of the whole extraction process. At this stage, a significant amount of methane remains inside the nanopores in the kerogen, but the extraction rate is very small. The pore network connectivity can significantly affect the ultimate recovery. It is important to note that the change in gas pressure is the main factor driving the transition from the first stage to the second stage for gas extraction. The transition point of gas pressure is around 20 atm.

Two-stage methane release revealed from MD simulations has important implications to the analysis of long-term shale gas production. Our work indicates that the long-term production is expected to be determined by the second stage of gas release. As discussed above, the dominant mechanism for gas release at this stage is methane desorption and diffusion in kerogen nanopores. The long-term production thus highly depends on the gas adsorption/desorption isotherm (e.g., the adsorption capacity) and the nanopore structures (e.g., pore connectivity) of shale matrix. These properties must be taken into account in a long-term production analysis. Our work also implies that the historical production data from the early stage of gas extraction may not provide sufficient underlying information for the prediction of long-term wellbore production.

Our work may help to understand the *b*-factor issue related to production decline curve analyses using Equation (1) as discussed at the beginning of this paper. We used Equation (1) to fit the calculated gas extraction rate curve (Fig. 3D). The averaged extraction rate was calculated over 9 samples as shown in Fig. 3C. Fitting the averaged extraction rates at different times using Equation (1) results in different *b*-factor values. Increasing the amount of historical data (i.e., increasing the total time interval) in the fitting leads to a decrease in the estimated *b* value. For instance, the *b* value deceases from ~1.2 to ~0.7 when the fitting time interval increases from ~110 ps to 1000 ps. The *b* value is not time-invariant. It must be made clear that, by no means, in this fitting analysis, we want to directly compare the calculated extraction rate curve with any actual field production decline curve because of their vast differences in both time and spatial scales and because of multiple maco-scale factors (e.g., micro-fracture networks) being excluded in our model. Nevertheless, this analysis raises a question about the validity of applying a single expression such as Equation (1) to a decline curve analysis over the entire time interval, because such a method may fail to capture the two-stage nature of the process. A reliable decline curve analysis calls for new model development based on mechanistic understanding of gas disposition and release in shale matrix. In addition, such mechanistic understanding may guide the development of new stimulation technologies for the extension of life cycle of a production well.

## Methods

### Formation of condensed kerogen

To create the condensed kerogen as shown in Fig. 1B we conducted a series of MD simulations^30^. Initially, we simulated 24 kerogen molecules in a box of 10 × 10 × 10 nm^3^ using NVT ensemble (1000K) for 100ps. During this simulation we collected one kerogen snapshot every 10ps. The 9 collected configurations were then compressed in NPT simulations in which the pressure was kept at 100 bar and temperature was gradually reduced to 900, 700, 500, and 300 K (100 ps for each temperature). At the last step, we reduced the pressure from 100 bar to 1 bar while keeping the temperature at 300K in the 100 ps NPT simulations. The final kerogen samples (9 samples) under room conditions were collected for characterization.

### Adsorption isotherm of methane on kerogen

To calculate total uptake we conducted grand-canonical Monte Carlo simulations (GCMC). The principle of this method is that the gas in the kerogen is in equilibrium with the gas in an imaginary reservoir. One of the inputs for GCMC simulation is the methane chemical potential. The output from the GCMC simulations is the number of gas molecules in the kerogen as a function of the chemical potential. This output cannot be directly compared with experimental data because the chemical potential cannot be measured. Therefore, we performed “empty box” simulations (i.e., a box without kerogen) to establish the gas pressures at specific chemical potentials^47^. Another approach would be to calculate the gas pressure corresponding to a given chemical potential using an equation of state^48^.

Methane molecules were modeled using the united atom TRaPPE force field^49^. Interactions between methane and kerogen atoms were described using a L-J potential with the cutoff distance of 12 Å. Equilibrium was obtained when the number of methane molecules found in the kerogen sample reached a constant value. This number describes the total uptake. The excess adsorption was estimated using the following equation: *n*_*excess*_ = *n*_*total*_−ρ_*P,T*_*V*_*free*_ where *n*_*excess*_ and *n*_*total*_ are the excess adsorption and total uptake, respectively, and ρ_*P,T*_ is the density of methane calculated using the Peng-Robinson equation of state^50^ at a specific temperature and pressure. *V*_*free*_ is the pore volume in kerogen determined by conducting GCMC simulations for non-adsorbed He^51^ at a low pressure and temperature.

### Extraction of methane from micropore in kerogen

To extract the methane from the kerogen we conducted MD simulations in the NVT ensemble (T =338K) starting from the configuration obtained from GCMC simulations at gas pressure of 262 atm, combined with the “fix evaporate” procedure available in the special package MISC of LAMMPS^52^. When the “fix evaporate” procedure is used, the temperature of the system, which is dependent on the number of atoms present, needs to be recalculated after each extraction step. For the evaporate procedure, we defined a spherical region of radius 3 Å within a kerogen pore. When methane molecules move into this region, they are deleted or removed from the system. We preset the maximum deletion rate at 5 molecules for every 500 time steps (i.e., 0.5 ps). Our reported extraction rates depend on the number of methane molecules that move into the defined region during a 0.5 ps timeframe and are always smaller than our preset maximum deletion rate. To evaluate the impact of the size of our extraction sphere, we performed an additional simulation using a sphere with a 5 Å radius. The difference in the extraction rate profiles for the 3 Å and 5 Å spheres is insignificant (Figure S3). In addition, because all the molecules moving into the extraction region are withdrawn, the region is effectively a vacuum. Thus, our simulation setup is analogous to the constant pressure (bottom borehole pressure) constraint used for field production.

The number of methane molecules remaining in kerogen was recorded as a function of time to calculate the extraction rate and monitor the gas pressure. In general, it is very difficult to calculate the gas pressure in the narrow space. We estimated gas pressure by combining the results obtained from the extraction simulation and GCMC simulation. In particular, in the extraction simulation we recorded the number of gas molecules remaining inside kerogen as a function of time. In the GCMC simulation, we obtained the number of gas molecules inside kerogen as a function of pressure. By combining these data we infer the pressure as a function of time in the extraction simulation as reported in Fig. 3B.

## Additional Information

**How to cite this article**: Ho, T. A. *et al*. Nanostructural control of methane release in kerogen and its implications to wellbore production decline. *Sci. Rep.* **6**, 28053; doi: 10.1038/srep28053 (2016).

## Supplementary Material

Supplementary Information
