# Supplementary material for: Nanostructural control of methane release in kerogen and its implications to wellbore production decline
Source: Sci Rep. 2016 Jun 16;6:28053. doi: 10.1038/srep28053 (PMC4910085; doi:10.1038/srep28053)
Supplement: Supplementary Information [file srep28053-s1.pdf]

Nanostructural control of methane release in kerogen and its implications to wellbore production decline

Tuan Anh Ho[1], Louise J. Criscenti[1*], and Yifeng Wang[2]

[1] *Geochemistry Department, Sandia National Laboratories, Albuquerque, New Mexico 87185, USA.*

[2] *Nuclear Waste Disposal Research and Analysis Department, Sandia National Laboratories, Albuquerque, New Mexico 87185, USA.*

Corresponding author

**Louise J. Criscenti, Sandia National Laboratories, Albuquerque, NM 87185, 505-284-2339, ljcrisc@sandia.gov*

Supporting Information

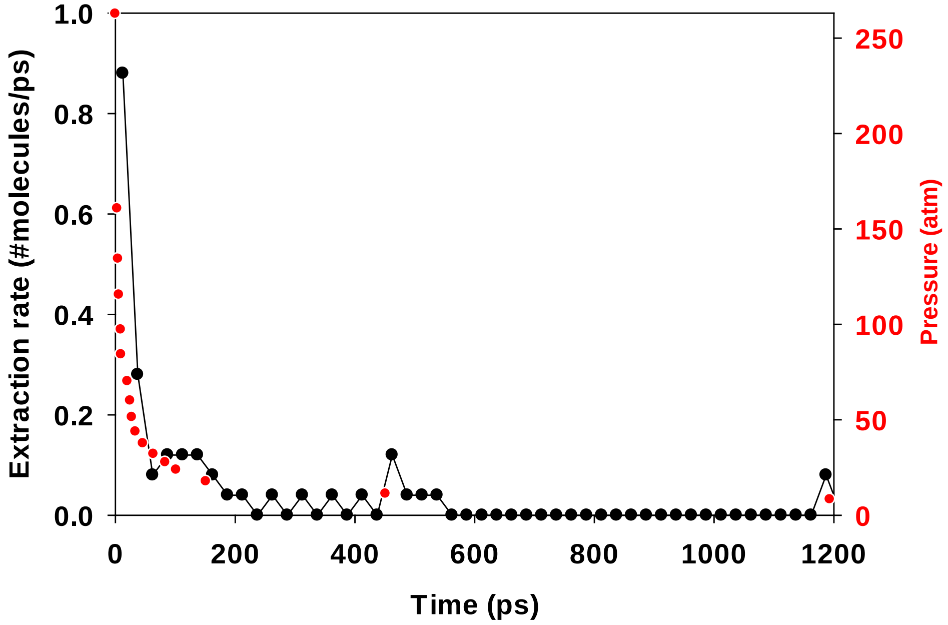

Figure S1. Extraction rate (black circles-left y axis) and pressure (red circles-right y axis) as a function of time during the extraction calculated for kerogen sample 2.

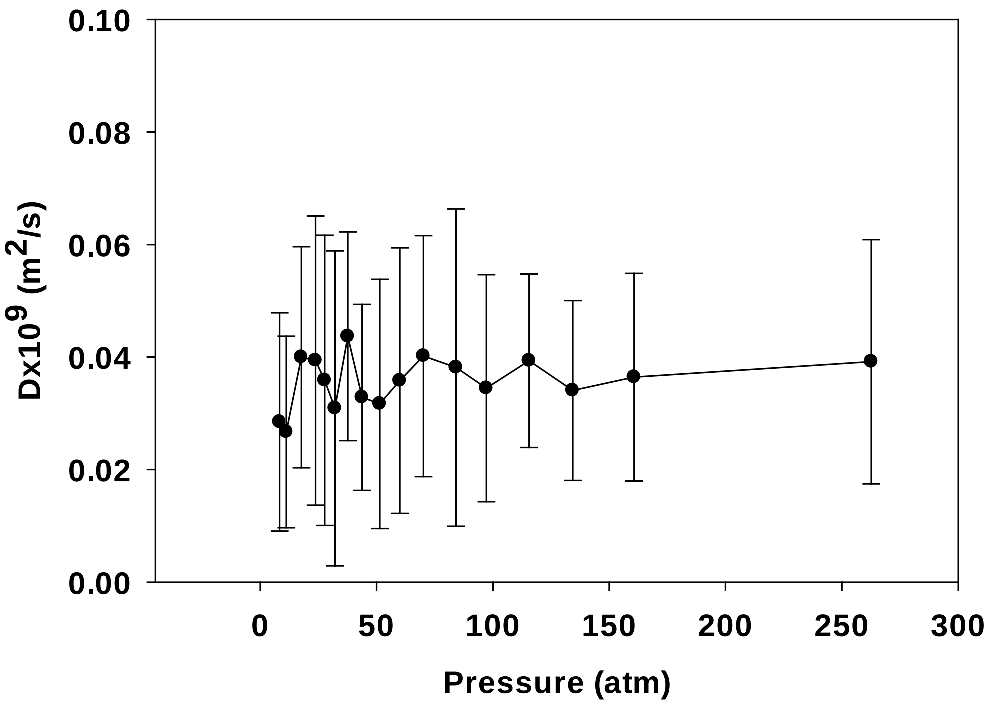

Figure S2. Self-diffusion coefficient of methane inside kerogen as a function of pressure during the extraction calculated for kerogen sample 2.

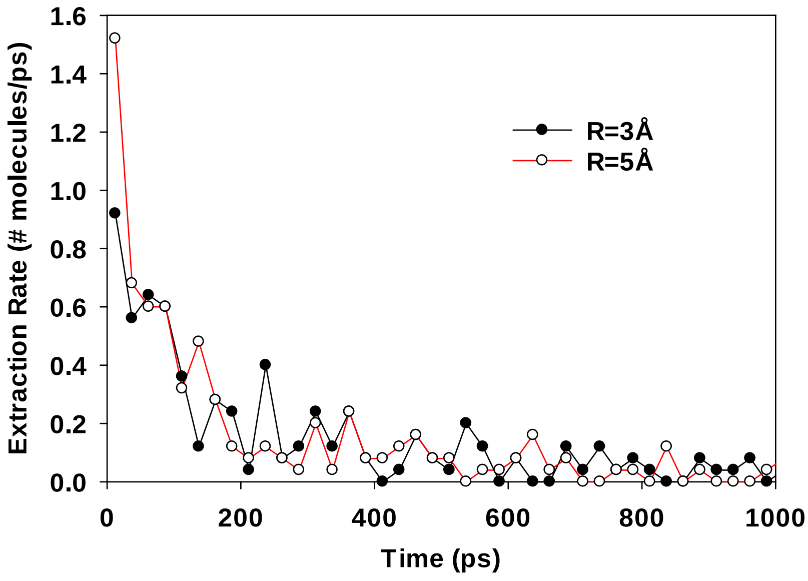

Figure S3. Extraction rate as a function of time when the radius of the extraction region is 3Å (black) and 5Å (red).
